# How Different Dimensions Shape the Definition of Meat Alternative Products: A Scoping Review of Evidence between 2000 and 2021

**DOI:** 10.1016/j.cdnut.2023.101960

**Published:** 2023-06-08

**Authors:** Linsay Ketelings, Remco C. Havermans, Stef P.J. Kremers, Alie de Boer

**Affiliations:** 1 Food Claims Centre Venlo, Campus Venlo, Maastricht University, Venlo, The Netherlands; 2 Laboratory of Behavioural Gastronomy, Centre for Healthy Eating and Food Innovation, Maastricht University Campus Venlo, The Netherlands; 3 NUTRIM, Department of Health Promotion, Maastricht University, Maastricht, The Netherlands

**Keywords:** meat substitutes, meat analogs, plant-based meat, plant-based foods, alternative protein

## Abstract

Consumer awareness of meat-associated health and environmental risks is increasing and motivates a shift toward consuming meat alternatives. This is also reflected in efforts invested in studying meat alternatives from the perspective of nutritional, environmental, and consumer sciences. Despite shared research interest, these studies cannot be readily compared and interpreted because there is no clear consensus on what meat alternatives are. Scholarly debates on acceptance, nutritional value, and environmental advantages of meat alternatives would benefit from a clear definition of meat alternatives. With the goal of defining meat alternatives, relevant scientific literature in the past 10 years was systematically searched and screened guided by the scoping review Preferred Reporting Items for Systematic Reviews and Meta-Analyses extension. The initial search resulted in >100,000 hits, which was reduced to 2465 papers. Next, titles and abstracts were scrutinized using Rayyan.ai, resulting in 193 articles considered for the present review. Article screening and data extraction was performed using ATLAS.ti software. Three major themes were identified to define meat alternative products including: *1*) producing and sourcing of ingredients; *2*) product characteristics (that is, sensory characteristics, nutritional value, and health profile, social and environmental sustainability profile); and *3*) consumer characteristics concerning the marketing and consumption context. Meat alternatives are multifaceted, that is, certain products can be considered as meat alternatives in some context, but not in another context. For any product, it is impossible to unequivocally state that it is a meat alternative. There is a lack of consensus from the diverse literature on what constitutes meat alternatives. However, products may be qualified as meat alternatives according to three key criteria as proposed in a taxonomy: *1*) production and sourcing, *2*) product characteristics, and/or *3*) consumption. We recommend researchers (and other stakeholders) to do so as it allows for better informed future discussions of meat alternatives.

## Introduction

The global population is expected to reach 9.7 billion people in the year 2050 according to projections of the United Nations [1]. This comprises an increase of ∼2 billion people in the coming three decades. The projected population growth imposes notable pressure on current resources, such as materials, energy, and food [2]. A particular nutrient associated with global population growth concerns the demand for animal-based protein. An increased demand for animal-based protein (such as meat and dairy) has been observed over the last decades and is expected to rise further [3]. Meat consumption is projected to increase because of a rise in the population. Not only is the role of protein in a healthy diet is increasingly recognized [4] but it is also expected that a larger proportion of people will be able to afford animal-based protein because of globalization and economic growth [5,6]. This projected global rise of animal-based protein demand, however, may have serious negative consequences in terms of both public health and the environment.

### Drawbacks of meat consumption

Production and consumption of animal-derived food products (including meat) have a strong environmental impact, while also creating an unacceptable animal welfare situations and working conditions. Employees (often migrant workers) in the meat processing and packing industry often work long days in crowded meat plants and receive minimal wages, possibly creating consequences for human rights [7]. The livestock sector is responsible for 16.5% of the global greenhouse gas emissions and has high land and water use [8]. Clark et al. [9] found that even with eliminating all fossil fuel, the current food production systems would prevent reaching the Paris Agreement’s Goal of limiting the global temperature increase of 1.5–2.0°C. It is therefore encouraged by organizations such as the Intergovernmental Panel on Climate Change to reduce consumption of animal products to improve public health and the environment [10,11]. Large-scale industrial meat product has consequences for many aspects of sustainable diets and food systems (that is, human health, ecological health, social equity and economic prosperity) [5,7]. Alternatives to meat can replace traditional animal-sourced meat products and thus address the challenges of the current food system.

The body of evidence about adverse health effects of particularly red processed meat consumption is expanding. A meta-analysis of prospective studies by Larsson and Orsini [12] showed that consumption of processed red meat is positively associated with all-cause mortality. Although Qian et al. [13] recommend more research into health effects of processed and red meat, the epidemiological data currently published are consistent. It indicates increased risks for the development of type 2 diabetes, cardiovascular diseases, and cancer (in particular colorectal cancer associated with the consumption of processed and red meat). Nevertheless, the health impact of meat consumption may vary for different types of meat, and may vary by the nature of processing, age group, and other factors. Dietary guidelines globally are being altered with the aim to reduce the consumption of processed red meat, but it appears that people are generally unwilling to change meat consumption habits despite health concerns associated with meat consumption [14].

### Drivers of meat production and consumption

Current and future projected meat consumption patterns have been linked to negative consequences for health, environment, and animal welfare. So why eat meat at all? Meat is an important source of protein [15]. It is a nutrient-dense food product, providing a relatively large amount of nutrients in relation to the energy content [16]. Meat contains all essential amino acids and several micronutrients such as vitamin B12, iron, and zinc. The bioavailability of nutrients in meat is high when compared with the availability of the same nutrients in foods derived from plants [17]. But meat is not just consumed for its nutrients. The smell, look, and taste of meat (and meat products) are often the main reasons for people to incorporate it in their diet [18].

Meat eating is also rooted in different cultural traditions around the world [19]. Furthermore, eating a hot dog in the United States, enjoying chorizo or cured ham as a tapas dish in Spain, Korean barbecue, or ordering a “frikandel special” in the Netherlands contributes to one’s identity. Indeed, certain meat dishes are consumed to adhere to a perceived national, sociocultural norm [20]. Despite meat consumption being attributed to certain occasions, many consumers also eat meat out of habit [21,22]. During upbringing, some people become accustomed to eating meat with every meal and, hence, meat consumption is not just a descriptive norm but also a dietary habit that is rarely reflected upon.

### Switching to meat alternative products

Meat is still an important component of the Western diet, but the availability of (plant-based) meat alternatives is rapidly increasing in response to an increasing demand. From 2018 to 2020, the market share of the European plant-based meat sector grew with 68% and is expected to grow over the upcoming years [23].

As a result of the rising popularity, research into meat alternatives is increasing too. Meat alternatives research involves different research disciplines examining, for example, the healthiness of meat alternatives, consumer beliefs regarding meat alternatives, and meat alternatives production methods [[24], [25], [26]]. Scientific studies on meat alternatives are important but still lack consistent terminology. Throughout the published literature, meat alternatives are sometimes referred to as meat substitutes, meat replacements, meat analogs, mock meat, or fake meat. It is not always clear whether these latter terms serve as synonyms for meat alternatives or are used to differentiate between proposed types of meat alternatives. The lack of criteria that clearly define a product as meat alternative makes it difficult for researchers, policymakers, and other stakeholders to review and evaluate the (dis-)advantages of meat alternatives. Moreover, in the absence of a definition, any research on meat alternatives can be contested and reduced to a semantic dispute.

### Aim of research

The aim of the current scoping review was to identify and characterize dimensions of nonmeat food products—intended for human consumption to support a healthy diet—that contribute to it being qualified as a meat alternative. Presumably, plant-based alternatives or alternatives containing egg or dairy protein compared with more traditional animal-sourced meat products. In doing so, we also aimed to create a taxonomy for meat alternatives to prompt the wider research community to adopt a sharper definition of what they consider to be a meat alternative. The current scoping review was thus guided by the question: “How are meat alternatives defined in recently published research (2000–2021)?” A scoping review was deemed the most appropriate research approach as it aims to “map the literature on a particular topic or research area and provide an opportunity to identify key concepts; gaps in the research; and types and sources of evidence to inform practice, product standards, policymaking, and research” [[27], [28], [29]].

## Methods

For this scoping review, the PRISMA extension for scoping reviews (PRISMA-ScR) and the methodical framework of Arksey and O’Malley [28]—advanced by Levac et al. [30]—were used to guide the review process [31].

Before conducting the literature search, a scoping review protocol was created detailing the research question, eligibility criteria, sample search strategy, study selection process, and data extraction criteria available at Open Science Framework (https://osf.io/an8ts/?view_only=8651835396c841e495f14476b0ef73fd).

### Data sources and search strategy

To cover a broad range of research disciplines, the following electronic databases were selected for the literature search: ScienceDirect, PubMed, HeinOnline, and Web of Science. Through a systematic keyword search in April and May 2021, the databases were searched for relevant literature.

The first search query consisted of the following search terms: (“meat alternative∗” OR “meat analog∗” OR “meat substitute∗” OR “meat replacement∗” OR “artificial meat∗” OR “mock meat∗” OR “fake meat∗” OR “hybrid meat” OR “plant-based meat∗” OR “plant-based meat alternative∗” OR “blended meat∗” OR “synthetic meat∗” OR “plant-based protein∗” OR “plant-protein∗” OR “insect∗” OR “insect-based protein∗” OR “insect protein∗” OR “entomophagy” OR “algae” OR “pea∗” OR “pulse∗” OR “legume∗” OR “cultured meat” OR “in-vitro meat” OR “cultivated meat” OR “lab-grown meat” OR “cell-based meat” OR “synthetic meat” OR “clean meat” OR “cellular agriculture” OR “alternative protein∗”). The first full search strategy and number of results for each database is shown in Supplemental Table 1.

### Eligibility criteria

All English language literature sources with a primary focus on meat alternatives (or synonymous) were included in the scoping review. Research into this field has garnered considerable attention only in the last decade (as shown previously [[32], [33], [34]]). Therefore, the literature search was limited to publications from 2010 onwards. Publication type, publication year, and language were not restricted until the screening stage, along with several search terms that yielded an inoperable number of results. To curtail the high number of results of the initial literature search, the following search terms were excluded for their broader scope because they did not address meat alternative products specifically: plant-based protein, plant-protein, insect, insect-based protein, insect protein, entomophagy, algae, pea, pulse, legume, cultured meat, in-vitro meat, cultivated meat, lab-grown meat, cell-based meat, clean meat, cellular agriculture, alternative protein. The final search strategy and number of results for each database are shown in Supplemental Table 2.

### Study selection, data charting, and analysis

Upon completion of the literature search, the results were imported into the web-based systematic screening software program Rayyan (Qatar Computer Research Institute). Duplicates were removed before the screening process. Two authors (LK and AdB) independently screened the titles and abstracts of the search results on relevance and eligibility criteria. Throughout the screening process the reviewers met to discuss any disagreements and resolve conflicts. If consensus could not be reached, another independent reviewer was consulted (RCH and SPJK) [30]. Subsequently, LK and AdB independently performed full article screening and data extraction of the included literature using ATLAS.ti (Version 8.4.5; Scientific Software Development GmbH).

In this second level of screening, all articles that did not discuss meat alternatives in detail were also excluded and all articles that did discuss meat alternatives were included. A set of 25 randomly selected articles was used to promote mutual understanding in the review process. Results of the data extraction and critical appraisal were discussed with the research team and any disagreements were solved through consensus.

**FIGURE 1:**
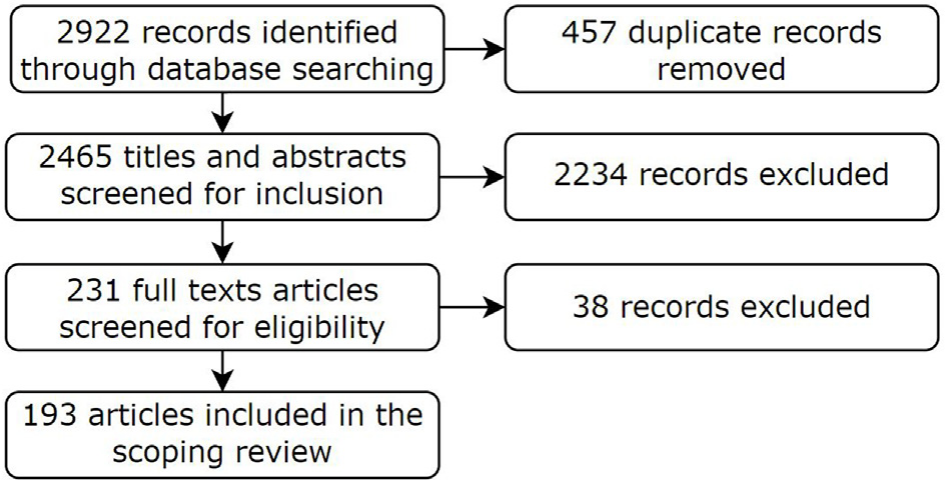
Flowchart of the study selection process (PRISMA-ScR flow diagram).

In Figure 1, the PRISMA-ScR flow diagram is presented. The initial search with the final search terms, as described in Supplemental Table 2, yielded 2922 records. After duplicate removal (*n =* 457), 2465 titles and abstracts were screened for inclusion. A total of 2243 records were excluded for the following reasons: *1*) neither the title nor the abstract indicated that meat alternatives were discussed in the given article (*n =* 712), *2*) the article only discussed a specific ingredient of meat alternatives (*n =* 482), *3*) the article appeared to be out of scope for this scoping review (for example, discussing meat reducing strategies) (*n =* 404), *4*) the article discussed only meat and not meat alternatives (*n =* 348), *5*) the article discussed technologies used when producing meat alternatives (*n =* 193), *6*) the article had the wrong publication type (for example, index or letter) (*n =* 88), *7*) the title and/or abstract was written in a language other than English (*n =* 5), or *8*) the article was published before 2010 (*n =* 2).

Of the remaining 231 included records, another 38 records were excluded after full text screening for the following reasons: *1*) the article was written in a language other than English (*n =* 15), *2*) the article did not address meat alternatives (*n =* 12), *3*) the full text was not available (*n =* 8), or *4*) the article was retracted (*n =* 3). A total of 193 articles were thus included in the present scoping review.

Information extracted from the included articles included were publication details (authors, year, journal) and all written information on what is understood by the authors as a meat alternative, which included specific definitions or characteristics of meat alternatives. Analysis of the extracted information entailed an iterative process of categorization to what meat alternatives (should) entail according to the authors. The iterative approach allowed us to create a model that is subject to continuous modification and revision throughout the course the review [35]. The analysis was done by labeling the relevant sections about meat alternatives of the included literature in ATLAS.ti.

Two reviewers independently assessed the literature and extracted definitions from the included literature, trying to prevent researcher bias and an objective approach to data analysis. The statements and definitions of meat alternatives presented in the result section display representative interpretations of the literature in a narrative format.

## Results

### Characteristics of literature

This review identified 193 sources of research in which meat alternatives are discussed. The general characteristics of the included studies are presented in Supplemental Table 3. Of the eligible sources, there were 96 original research papers, 46 reviews, 12 law reviews, 11 book chapters, and 28 academic sources falling under other types of literature (see Table 1). The majority of the literature is published in the field of consumer sciences (*n =* 77). Other fields of research include nutritional sciences (*n =* 42), food technology (*n =* 39), and environmental sciences (*n =* 34) (Table 1). What stands out from the literature characteristics is that the larger part is published in the United States (*n =* 46), The Netherlands (*n =* 32), and United Kingdom (*n =* 19) which are Western countries, whereas only little is published in Asia: South Korea (*n =* 9), India (*n =* 3), and Singapore (*n =* 3). An overview of countries of publication can be found in Supplemental Table 4.

**TABLE 1:** Overview table of article types and fields of research

Article type	%	Field of research	%
Original Research Article	49.7	Consumer sciences	31.6
Review Paper	23.8	Nutritional sciences	17.2
Law Review	6.2	Food technology	16.0
Book Chapter	5.7	Environmental sciences	13.9
Science Communication	2.1	Food law	5.3
Systematic Review	2.1	Sensory sciences	4.5
Abstract	1.6	Behavioral sciences	4.1
News Article	1.6	Business and economics	3.7
Opinion Paper	1.6	Social sciences	2.0
Commentary	1.0	Philosophy	0.8
Feature Article	1.0	Food safety	0.4
Magazine Article	1.0	Psychology	0.4
Editorial	0.5		
Poster Abstract	0.5		
Protocol	0.5		
Reference Module	0.5		
Short Communication	0.5		

### Production and sourcing of meat alternatives

The first characteristic that is identified from the included literature is that the production and sourcing of the meat alternative is a major determinant of what the product eventually looks like. The choices made during the production of a meat alternative, such as which ingredients are used and how these ingredients are processed, influence the appearance of the meat alternative.

#### Ingredients used in meat alternatives

A wide range of ingredients can be used to produce meat alternatives. Ingredients can be used for different purposes, namely for their sensory characteristics, functional properties, and to provide nutrients such as protein. Commonly found protein sources in meat alternatives are soy, wheat, mycoprotein, and lentils and pulses, which often make up the largest portion of the product. Soy protein (possibly in the form of textured soy protein) is one of the most used sources of protein in meat alternatives. It has valuable functional properties when creating a meat-like structure, and offers a balanced amino acid composition compared with other ingredients [36]. Cereal ingredients such as wheat are generally much higher in carbohydrate content and lower in protein content, and much lower in protein content compared with soy [37]. Next to plant-based sources of protein, also animal-based ingredients are sometimes used in meat alternatives, for example, egg, milk, or cheese [38].

Novel plant-based proteins such as seaweed, algae, or other single-cell proteins are gaining more recognition because of their nutritional properties [24]. Also, alternative proteins that are being increasingly acknowledged in literature are proteins derived from insects. Insects can provide similar nutrients to meat and require fewer resources to grow and process [25,[39], [40], [41]]. Another development in the field of meat alternatives is cultured meat. Cultured meat is animal meat produced with the help of tissue engineering techniques [42], except it is still up for debate whether cultured meat should be considered meat or a meat alternative [43].

Where protein makes up ∼20%–50% of the meat alternative, other ingredients are added as well for different purposes such as texture, taste, and nutritional value. In a review by Boukid [36], it is shown that next to protein, lipids, polysaccharides, flavoring and coloring ingredients, and fortifying ingredients are added to enable a meat-like experience [36]. Further development of novel ingredients will allow even higher quality meat alternatives, for example with improved sensory characteristics or nutritional value.

#### Production process of meat alternatives

As described in the previous section, the ingredients of meat alternatives often require some degree of processing. One type of ingredient concerns unprocessed and minimally processed primary agricultural ingredients such as (canned) pulses (beans, lentils, and peas), nuts, and grains [44,45]. A second type of ingredient is a more processed form of these primary agricultural ingredients, such as tofu and tempeh. A third type comprises ultra-processed ingredients such as textured vegetable proteins, mycoproteins (as used in Quorn), and other isolated proteins (for example, isolated soy protein) [45,46]. These different types of ingredients can be the source for a meat alternative product, and thus Apostolidis and McLeay [47] state that “Meat substitutes, …, are usually derived from soybeans (Tofu), algae and dairy products (e.g., Valess), plant proteins (Ojah) and mycoprotein (Quorn) which are sold as burgers, stir fry cubes and as mincemeat and resemble the taste and texture of meat.”

Although not all alternatives to meat that can be found in the supermarket are highly processed (tofu, for example), many of them are. The meat alternatives that intend to mimic a particular meat product can often be classified as ultra-processed food products [45,48,49]. On one hand, there are meat alternatives that contain less-processed proteins but do not mirror the experience of eating meat, and however, there are highly processed products to create a sensory experience (that is, appearance, mouthfeel, and taste) similar to meat [36]. Several scholars have expressed their concern regarding this ultra-processed nature of meat alternatives, because ultra-processed food products are typically products with low nutrient density that could be perceived as a health barrier to nutritionists and other stakeholders involved [37,45,[50], [51], [52]]. Nevertheless, some plant-based ingredients require heavy processing to produce a functional and edible product. It remains to be determined whether ultra-processed meat alternatives might serve as a healthier choice or improve diet quality, despite being potentially lower in nutrient density compared with meat.

### Product characteristics of meat alternatives

The choice of ingredients, the production process, and potentially the respective costs of meat alternatives determine the sensory characteristics, nutritional value, and healthiness of a meat alternative product. It also determines how sustainable the meat alternative is. These 4 aspects are also evident and elaborately discussed in scientific literature.

#### Sensory characteristics

An important facet when defining meat alternatives is the sensory characteristics of a meat alternative. Many authors state that a meat alternative must be “meat-like” and that its organoleptic properties must resemble meat. These organoleptic properties include texture, flavor, appearance, smell, and taste. An exemplary definition by Bakhsh et al. [53] underlines the importance of the sensory characteristics of meat alternatives: “… it possesses texture, mouthfeel, taste, and nutritional qualities that resemble meat.” In addition, Boots et al. [54] state that “… the product must mimic the aesthetic qualities of meat such as size, appearance, taste and texture.” The fact that meat alternatives should mimic meat to be accepted by consumers is also highlighted by several authors [36,43,[55], [56], [57]]. Put bluntly, if a meat alternative does not look, feel, smell, and taste like meat, it is not accepted as a meat alternative by most consumers [24].

To the contrary, some scholars emphasize that meat alternatives do not necessarily have to resemble meat. For example, in a review by Santo et al. [46], it is mentioned that a variety of meat alternatives exist. These include for example more natural alternatives resembling characteristics of meat (for example, jackfruit), products that are not designed to mimic meat (for example, tofu and tempeh), and more processed alternatives that are designed to mimic meat products.

#### Nutritional value and health

According to several authors, meat alternatives should match their equivalent meat products not only sensory wise, but also in nutritional value [37,[58], [59], [60], [61]]. With the currently available ingredients and production processes, this rarely appears to be feasible. Compared with meat, meat alternatives sometimes are found to be higher in sodium, higher in sugar, and lack micronutrients such as vitamin B12 and iron [52,[62], [63], [64], [65]]. A supermarket audit of plant-based meat alternatives by Curtain and Grafenaur [65] in Australia revealed that products have wide nutrient ranges and overall higher levels of sodium. Only 24% of the available alternatives were fortified with vitamin B12 and a mere 20% were fortified with iron. This is often considered as a shortcoming and numerous literature sources thus emphasize the importance of nutritional value in meat alternatives. Lee et al. [66] for example state that “It is very important to manufacture plant-based meat analogs to meet the nutrient specifications of traditional meat.” The fact that current meat alternatives fall short regarding the macro- and micronutrient composition compared with meat is concerning to some authors, especially with the current market growth in mind [58,65,67,68].

On average, a meat alternative product contains ∼20%–50% protein [36]. The composition of proteins and amino acids present in meat alternatives should be comparable with meat, according to some authors; especially because meat alternatives are sometimes referred to as “alternative proteins.” For example, Choudhury et al. [58] define meat alternatives as follows: “Plant-based meat alternatives are a sustainable source of protein that can match the taste and texture, color, and nutritional profile of specific types of meat.” In addition, it was observed by De Marchi et al. [69] that bioaccessibility and bioavailability of nutrients in plant-based foods should be carefully considered to correctly estimate the nutritional value and to properly compare them with their meat counterparts. The fact that little has been published regarding the nutritional composition and bioavailability of nutrients in meat alternatives could create difficulties in the interpretation of research results.

The health perception of meat alternatives is divided among consumers and researchers [70]. Meat alternatives could benefit health by reducing antibiotic use and lessening the likelihood of foodborne illness, however, they can impair health due to their highly processed nature [51,52,71]. In a review by Bohrer [37] where nutrient specifications of meat analogs were compared with traditional meat products, it was concluded that meat analogs can approximate the nutritional composition of meat but not without a high degree of processing. This makes it difficult to definitively state what product type (meat or meat alternative) is healthier.

#### Sustainability

A prominent argument for consumers to choose meat alternatives over meat is sustainability. Meat alternatives are proposed as one of the solutions to improve global environmental conditions because it requires fewer animals (that is, reduced livestock) for our future dietary needs. A decreased need for animal products promotes the reduction of greenhouse gas emissions [72]. Furthermore, a lot of land is now used to grow food for feeding livestock. Reducing the demand for meat would limit the need for expanding cropland that presently poses a threat to the conservation of biodiversity [72].

There appears to be a wide consensus that meat alternatives are in principle more sustainable than meat products. However, there is some speculation about whether meat alternatives are truly an environmentally friendlier option than meat because meat alternatives are very often classified as ultra-processed foods [58]. This processing burden requires extra energy that will contribute to carbon emissions. At present, it is sometimes unclear how much more carbon emissions are associated with the production of meat alternatives. Thus, it requires future in-depth research. But for the present discussion, one could argue that—at least for some consumers—a meat alternative would not really be an alternative to meat if it is not more sustainable than meat.

In a real-life setting (for example, on a label or near the shelves in the supermarket), often no or little information is provided regarding the environmental impact of a product. Consumers have to rely on their own knowledge and intuitive judgment to assess whether a meat alternative is more sustainable than meat [73]. In a review by Onwezen et al. [33], it is shown that consumers do consider the consumption of meat alternatives (including pulses, algae, insects, cultured meat and plant-based meat alternatives) for environmental benefits. Consumers find sustainability important, and this motivates them to choose meat alternatives believing these products are ipso facto more sustainable than otherwise equivalent meat products. Without a definition (that includes standards for sustainability, labeling, and advertising) consumers cannot make informed decisions about meat alternative products.

### Expectations of meat alternatives

Meat alternatives can be consumed for different reasons, such as sustainability reasons, animal welfare reasons, health concerns, or out of curiosity [24]. Different consumer segments have different associations with, and therefore also different expectations regarding meat alternatives. This was for example shown in a study by Possidónio et al. [74], where 3 groups of consumers could be identified: *1*) committed meat eaters with an aversion to meat alternatives; *2*) meat eaters concerned with health issues, nutrition, and functional values of eating; and *3*) a third group expressing disgust toward meat tied with ethical concerns for animals. Building on this, Apostolidis and McLeay [47] conclude that meat substitution strategies should focus on specific consumer segments, instead of targeting the average consumer, because of the different preferences and expectations of each segment.

Next to different target consumers, another element to consider is the context in which a meat alterative is consumed. Is it a fast-food setting, at home, or another social setting? When assessing the healthiness and nutritional value of a meat alternative, Hu et al. [51] state that “these popular PBMA [plant-based meat alternatives] burgers are often consumed in fast-food settings where they may be placed on a refined grain bun with an array of toppings, served with French fries and even a sugary beverage. Thus, it is not possible to assume that substituting a PBMA patty for a beef patty improves overall diet quality.” This is in line with other authors who also acknowledge the importance of the context in which a meat alternative is consumed [46,75,76]. It is essential to consider that a meat alternative is often not eaten on its own, but as part of a dish, a meal, or a tradition [75].

Although many meat alternatives are targeted to consumers seeking to reduce their meat consumption, there appears to be some unwillingness to switch from meat toward meat alternatives. Consumers are often unaware of the environmental and health issues surrounding meat [33]. Also, consumers are unfamiliar and uninformed about meat alternatives, resulting in low willingness to consume meat alternatives [77]. It is not possible to convince consumers to switch to meat alternatives if they are not ready to try new products [24,78].

The expectations set by the target consumer and the intended consumption context of the product influence the product characteristics described previously in section “Product characteristics of meat alternatives”. Different consumers have different requests, needs, desires, and expectations when looking for meat alternatives relating to sensory characteristics, nutritional value, health, or sustainability. Therefore, the target consumer eventually decides what products can be considered a meat alternative, a decision that is influenced by prior expectations.

## Discussion

This scoping review was carried out with the aim to identify and characterize dimensions of a nonmeat food product that contribute to it being qualified as a meat alternative. A total of 193 literature sources were included in this study in which the authors were describing and defining meat alternatives. There is a large variety in types of sources and fields of science, indicating the diverse nature and scope of meat alternative research. This diversity in resources and fields of science led to different definitions about what is considered, or influences the characteristics of a meat alternative. Several dimensions could be discerned that inform the qualification of a product as a meat alternative, namely: the production process and ingredients, product characteristics of meat alternatives (including sensory characteristics, nutritional and health value, and sustainability), and consumer expectations. It should be noted that some of the included literature did not explicitly coin or describe these facets.

### The dimensions that define a meat alternative

There are several important aspects when it comes to defining a meat alternative. The present review shows that researchers into the field of meat alternatives do not have a shared understanding of what a meat alternative exactly is. A large portion of the authors of the included literature do not (or barely) define what a meat alternative is, such as “meat alternatives are typical products with respect to the initial liking, complexity and newness” [79]. In most articles, only some indirect definitions could be found, which underscores the relevance of the current scoping review.

When a definition of a meat alternative is provided, authors often consider meat alternatives as products that only have similar sensory characteristics to their meat equivalent with the goal of making more sustainable choices for consumers. Others also stress the importance of comparable nutritional value and health benefits. There are products available on the market that do not possess similar sensory characteristics like meat but are considered as an alternative to meat by consumers (for example, tofu, tempeh, and burgers made from [whole] vegetables or mushrooms). Also, within the categories of meat alternatives, there is a large variety regarding nutritional value, healthiness, sensory characteristics, and environmental impact. A meat alternative can thus serve different purposes in different consumption contexts, whether it is to replace the experience of eating meat or merely to replace the protein component, both still can be considered a meat alternative.

Hwang et al [80] pose that the goal of meat alternatives is to satisfy meat eaters (and not vegans) in terms of taste, texture, and appearance, whereas Circus and Robison [81] found that meat alternatives are favored mostly by low meat attachment consumer groups, such as people adhering to a vegetarian or vegan diet. Different consumer segments have different needs and hence expectations regarding what makes a meat alternative a true and appropriate alternative to meat. Some consumers might be troubled by consuming food that closely resembles meat [82], whereas others may prefer meat alternatives that closely approximate their meat-like counterpart [83]. For any given consumer, a meat alternative can be defined as a product with similar sensory characteristics, equivalent/superior nutritional value, or health benefits, and as a more sustainable alternative to meat. These varying attributes are in turn determined by (the processing of) certain ingredients [47,74,84]. In short, there is no single definition of a meat alternative.

Nonetheless, there is a definite set of dimensions that qualify meat alternatives, that is, if a product does not satisfy a single dimension, that food product is not a meat alternative and when all dimensions are satisfied the product is clearly a meat alternative. When one or only some dimensions are satisfied, the product may be seen and used as a meat alternative by some consumer segments. Identifying these dimensions then provides a structure that aids researchers, food business operators, and other involved stakeholders to carefully evaluate new products aiming to replace meat on the consumer’s plate.

### Taxonomical structure to define a meat alternative

A taxonomical structure can help researchers, food business operators and other stakeholders involved to critically think about what is considered to be a meat alternative on the different key dimensions as described in the results section.

**FIGURE 2:**
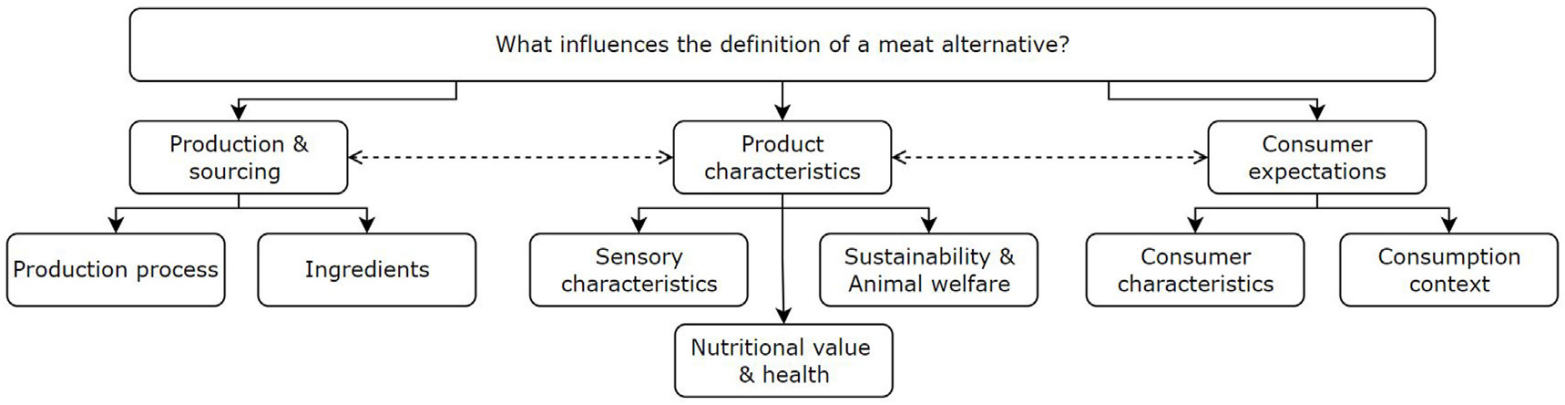
Taxonomy of the dimensions that define a meat alternative. Three facets are displayed that influence what defines a meat alternative. Importantly, the product characteristics displayed in this figure are influenced by the 2 other facets, being production and sourcing and consumer expectations. Both facets however also influence how these product characteristics are formed.

Figure 2 provides an overview of the facets that influence what defines a meat alternative. In this scoping review, three main dimensions are identified that influence the meat alternative definition, namely production and sourcing, product characteristics, and consumer expectations. Each of these main dimensions has several subdimensions, providing more depth into facets that influence the definition of a meat alternative. The dimensions production and sourcing and consumer expectations both interact with the product characteristics dimension. The food business operator could have certain wishes regarding: sensory characteristics, nutritional value and health, and sustainability that influence the production of meat alternatives. In turn, the production of a meat alternative and the sourcing of its ingredients influences what the sensory characteristics of a product are, what nutritional value is, and how sustainable the product is. To the contrary, the product characteristics influence the expectations a consumer can have, because marketing a product with certain sensory characteristics, for example, will influence these expectations. Furthermore, the expectations a consumer can have (based on its characteristics and the intended consumption context) can affect the product characteristics dimension. This dimension would also fit the costs a consumer pays for a meat alternative as well as how the products are marketed. Although this taxonomy is focused on meat alternatives only, it could be useful as well in defining other forms of alternative proteins, such as products produced through cellular agriculture and dairy and seafood alternative. Although all identified dimensions could be applicable to such products, it remains to be confirmed whether these dimensions are indeed of similar importance in other alternative protein categories.

### What’s in a name—meat alternatives

Throughout this paper, the term meat alternative is used, although many different synonyms are used among authors to refer to a similar product. For example, meat substitute, meat analog, and meat replacement. Regardless of the used synonym, in most cases, authors refer to a product with an appearance, texture, and taste similar to that of meat. Specifically, meat analogs and meat imitations are only referred to as products analogous to meat, thus, with sensory properties like meat. Plant-based meat alternatives are specified as products containing only ingredients from a plant-based origin, whereas other products could contain ingredients from animals such as milk, eggs, or cheese.

Synonyms such as meat alternatives, meat substitutes, and meat replacements are used interchangeably. Unless specified by the authors, it is difficult to determine what types of meat alternative products are actually meant: similar in sensory characteristics and nutritional value, or not per se.

Meat alternatives resembling meat and using meat-like terminology on the label and in marketing have been a point of discussion over the last years. In 2019, a proposal was drafted by Directorate-General for Agriculture and Rural Development (DG AGRI) [85] to the European Parliament to reserve the use of terms such as “burger,” “sausage,” and “steak” exclusively for products containing meat. In the EU, meat is extensively defined and regulated in food legislation. Regulation 1308/2013, establishing a common organization of the markets in agricultural products (also known as the COM regulation), defines “meat” as all carcasses, meat on the bone or boned, and offal, whether or not cut, intended for human consumption, obtained from bovine animals aged <12 months, presented fresh, frozen or deep-frozen, whether or not wrapped or packed [86]. In addition, Regulation (EC) 853/2004, laying down specific hygiene rules for food of animal origin, defines “meat” as the edible parts of animals [87]. Lautenschlaeger and Upmann [88] indicate that there is room for debate upon the definition of meat, but the current legislation is generally acceptable for stakeholders.

Although the term “meat alternative” has the word “meat” in it, the meat alternative that can be bought at retailers has no meat in it. Terms such as “sausage” and “burger” have no legal definition in the EU, but it has been argued that using these can indicate the product contains meat. The European Consumer Organisation (BEUC) [89] however put forward that using meat terminologies on the label of meat alternatives are not misleading plus they are useful to create consumer expectations. In October 2020, the proposed amendment was rejected by the European Parliament and therefore the use of meat-like terminology is currently allowed on meat alternatives.

Not only in the EU has the use of meat-like terminology on meat alternatives been up for debate. Also in the United States this was pointed out. Lawmakers in several states passed statutes that forbid the use of “meat” in the labeling of plant-based meat products. This was justified as protecting consumer choice and public health by preventing a confusing and misleading labeling scheme. Meat alternative companies argue that this is unconstitutional, because this prevents sharing “truthful information and impede competition” in the market and it is violating rights to commercial free speech [90]. Contrastingly, in December 2019, the Plant Based Food Association [91] published voluntary standards for labeling of meat alternatives in the United States. Here, they define a meat alternative as “A solid food produced mainly with plant-based ingredients that may have textural, flavor, appearance or other characteristics typically associated with animal-meat based products but that is free of meat from any animal.” In these voluntary standards, the Plant Based Food Association states that references to animal-based terminology (such as hamburger, sausage, chicken) are allowed if the label clearly indicates that the product is plant based or vegetarian.

Across different jurisdictions, the political debate surrounding labeling of meat alternatives has not settled yet. That debate would benefit from more research on what impact labeling and the marketing of meat alternatives with meat terminology would have on consumer behavior. The question remains whether referring to meat products in labeling and marketing meat alternatives is confusing, or misleading the consumer.

### Impact of labeling and marketing

Labeling does not necessarily influence the definition of what a meat alternative should comprise. The definition itself however does have an influence on how these products are labeled and marketed. In addition, the label determines for whom the product is intended (in other words, the target consumer) and sets expectations regarding sensory characteristics and the context in which the meat alternative is planned to be eaten.

Names that imply the product resembles meat, such as “burger”, “schnitzel,” and “sausage,” convey information that sets expectations for a specific consumer [92]. A product that is marketed as a “vegan sausage” should therefore have the characteristics of a sausage from animal origin. However, some uncertainties remain on what these characteristics should be. There is consensus that these products should at least have similar sensory aspects compared with their meat equivalent, but some unclarity regarding the nutritional values of the products prevails. Currently, most meat alternatives do not possess similar (or improved) nutritional values when compared with their meat equivalent, whereas some consumers do have this expectation [93]. It is namely argued by Curtain and Grafenauer [65] that labeling the products as a meat-like product sets the expectation that it mirrors the nutritional values, which could be considered confusing.

Next to the use of meat terminology on meat alternatives, the use of some other terminology may evoke certain responses in some consumers. The use of “tofu” and “vegan” has been found to evoke a negative response from consumers with an omnivorous or flexitarian diet, whereas the term “plant-based” appears to be more accepted among consumers [33,94]. To the contrary, people with a vegan diet are specifically looking for products that carry the label vegan [91]. The labeling on meat alternative packaging therefore also influences the target consumer that companies wish to reach.

### Practical implications

In the current market, the ideal meat alternative does not exist. There is not one meat alternative available that can match all the specific expectations of different consumer segments.

The largest consumer segment that consumes meat alternatives is people with an omnivorous diet looking to reduce their meat consumption without giving up the pleasure from eating meat [95]. Hence, they are looking for meat alternatives that closely resemble meat on mainly a sensory level. Characteristics such as taste, flavor, texture, smell, and appearance should match with the animal counterpart to be the most appealing for this consumer segment. In addition, meat is currently one of the main sources of protein in the human diet and the amino acid composition of it is valuable by providing essential amino acids [17]. Nevertheless, (processed) meat products such as ham, bacon, and salami are classified as a group 1 carcinogen by the International Agency for Research on Cancer of the WHO, which means there is strong evidence that processed meat consumption could lead to the development of cancer [96]. Therefore, next to being sensory alike, consumers also start looking for healthier alternatives to meat with a similar or even better nutritional value [97].

To the contrary, there are also smaller groups of consumers that prefer a meat alternative product that does not have similar sensory characteristics or do not really mind that the product is not resembling meat. It appears that when people more regularly consume meat alternatives, the less they want or need meat alternative products to resemble meat [83].

By considering the specific target consumer as a meat alternative producer, a product can be developed on the different product characteristics (sensory characteristics, nutritional value, health, and sustainability) according to expectations of a meat alternative. It is important for manufacturers to strive for meat alternatives that are animal friendly, nutritious, and healthy that are not distinguishable from meat at all.

Although the taxonomy in Figure 2 is currently focused on (plant-based) meat alternatives only, it might serve a purpose in defining other related products too. Other alternative protein products such as insects, hybrid products, and cultured meat could benefit from a carefully constructed definition as well. For cultured meat, for example, it could consider the different proposed themes in the taxonomy to answer questions concerning production, product characteristics and consumer expectations to determine what a cultured meat product should look like. Additional reviews of currently available literature are recommended to determine whether the proposed taxonomy includes all essential dimensions for constructing a fitting definition.

### Strengths and limitations

This is the first scoping review that aims to define a meat alternative and what it should entail. The current approach allowed us to focus on the state of research activity in the field of meat alternatives, rather than the quality of the literature and collects information on an emerging topic [98]. A systematic process was followed using the PRISMA guidelines for scoping reviews, with relevant and appropriate search terms to gather literature. Next to this systematic approach being a major strength, this scoping review will help future research and development of meat alternatives on an academic and industry level. However, some limitations should be acknowledged as well. First, the large amount of published literature imposed a strict selection of the search terms used in this scoping review. Consequently, some articles could be missing in the current set of literature. Another limitation is omission of a large subset of literature about cultured meat. Not considering cultured meats in the current review does not in any way disqualify these products as meat alternatives. It did allow for a more focused literature search and discussion. In addition, the iterative approach in the present review consisted of continuously reviewing data and translating these into a refinement of the model, which could be difficult to replicate. Because this field of research is moving fast, at the time of writing, additional literature on meat alternatives is published which could not be included in this scoping review. Nevertheless, with the thoughtful consideration of the search terms, it is expected that all facets on how to define a meat alternative have been discovered and described.

## Conclusions and outlook

The purpose of the current scoping review was to identify and characterize dimensions contributing to a product being qualified as a meat alternative. This research has shown that when defining a meat alternative, several dimensions need to be considered: production and sourcing of meat alternatives, the product characteristics that define a meat alternative (sensory characteristics, nutritional value and health, and sustainability), and expectations of meat alternatives by consumers. These expectations are determined by the characteristics of the target consumer and the consumption context. Marketing and labeling of meat alternatives require careful consideration by different stakeholders, because different consumers have different expectations of what their ideal meat alternative product should comprise. The taxonomy proposed should be tested and compared with other emerging classification systems for traditional animal-source and plant-sourced proteins that are evolving in response to the transition and transformation toward sustainable and healthy diets and food systems for the future.

It is recommended that stakeholders, including researchers, policymakers, and industry, make meat alternatives more accessible to the different consumer segments. These efforts will aid in reducing the detrimental effects of intensive animal farming on the earth’s climate. Future research and product development could focus on improving the dietary composition and nutritional quality of meat alternatives in comparison with meat, because there currently is a wide variety in and between different products in the supermarkets. This could be done by, for example, lowering product sodium levels or the addition of fiber. Functional foods are more appreciated by different types of consumers nowadays, which is also an angle that can be investigated in the near future. It is important that this fast-growing field of research will result in healthier, sustainable, and tasty products that are available for a bigger audience. A well-defined definition in research and development of meat alternatives is of significance to make clear choices in the best interest of the consumer as a food business, legislator, or policy maker.

## Funding

This research has been made possible with the support of the Dutch Province of Limburg, who had no role in the setup or conduct of this research.

## Author disclosures

The authors report no conflicts of interest.

## Data availability

Data described in this manuscript will be made publicly and freely available without restriction at https://osf.io/an8ts/?view_only=8651835396c841e495f14476b0ef73fd.

## Acknowledgments

We would like to thank Quinten de Bakker (VieCuri Medical Centre) for his assistance with the literature search.

## Author contributions

The authors’ responsibilities were as follows—LK, RCH, SPJK, AdB: design and methodology; LK, AdB: reviewing and analysis; LK: writing—original draft; RCH, SPJK, AdB: review and editing; AdB: primary responsibility for final content; and all authors: read and approved the final manuscript.

## Appendix A Supplementary data

Supplementary data to this article can be found online at https://doi.org/10.1016/j.cdnut.2023.101960.

## Appendix A Supplementary data

Multimedia component1 Multimedia component1
